# Structural characterization of the human CC2D1A fragment associated with non-syndromic intellectual disability (NSID)

**DOI:** 10.1042/BSR20253955

**Published:** 2026-05-21

**Authors:** Yi-Hung Yeh, Min-Guan Lin, Xing-Han Sun, Yo-You Shen, Pin Ling, Chwan-Deng Hsiao

**Affiliations:** 1 Institute of Molecular Biology, Academia Sinica, Taipei 115, Taiwan; 2 Department of Microbiology & Immunology, College of Medicine, National Cheng Kung University, Tainan 70101, Taiwan

**Keywords:** C2 domain, CC2D1A, DM14 domain, DNA binding, non-syndromic intellectual disability

## Abstract

CC2D1A is a multidomain scaffold protein implicated in transcriptional regulation and autosomal recessive non-syndromic intellectual disability (NSID), yet its molecular mechanism is still poorly understood due to a lack of structural information. Here, we present the crystal structure of the human CC2D1A_491–__810_ fragment, encompassing the fourth DM14 domain, a coiled-coil region, and a C-terminal C2 domain. These elements form a compact, integrated architecture, with the C2 domain mediating symmetric dimerization through conserved electrostatic interactions. In addition, a unique antiparallel β1–β10 sheet connects the coiled-coil and C2 domains, stabilizing the tertiary structure. Fluorescence polarization assays reveal micromolar DNA-binding affinity, likely mediated by the basic surface of the DM14 domain. Comparison with the *Drosophila* homolog Lgd highlights conserved topology with added structural features, offering insights into CC2D1A’s vertebrate-specific functions and NSID-related mutations.

## Introduction

CC2D1A (Coiled-coil and C2 domain-containing protein 1A), also known as Freud-1, Aki1, or TAPE, is a multifunctional scaffolding protein implicated in a variety of cellular processes, including transcriptional repression [1,2], Akt-mediated survival signaling [3], NF-κB activation [4], endosomal trafficking, Notch pathway regulation [5,6], and centrosomal cohesion during cell division [7]. These functions span both cytoplasmic signaling and nuclear transcriptional control, positioning CC2D1A as a central integrator of developmental and stress-responsive signaling. Loss-of-function mutations in CC2D1A have been identified as causative for autosomal recessive non-syndromic intellectual disability (NSID) [8], and *Cc2d1a* knockout mice exhibit perinatal lethality due to respiratory failure, highlighting its essential role in early neural development [9]. In addition to its role in NSID, CC2D1A has also been implicated in autism spectrum disorder (ASD) [10–12]. A large-scale exome sequencing study identified CC2D1A among ASD risk genes enriched in mid-fetal cortical development and assigned it to gene expression regulation modules based on co-expression analysis [13]. These findings suggest that CC2D1A contributes to ASD pathogenesis through transcriptional regulatory mechanisms active during early brain development.

CC2D1A comprises multiple main conserved domains, including four DM14 repeats, a central coiled-coil region (CC), and a C2 phospholipid-binding domain (C2). Each of these domains is associated with distinct molecular functions: DM14 motifs mediate interactions with CHMP proteins, nucleic acid, and phospholipids [2,6]; the coiled-coil region domain engages in sequence-specific DNA binding and transcriptional repression at conserved DRE elements [2]; and the C2 domain is required for lipid binding, subcellular localization, and NF-κB activation [4]. Beyond these biochemical roles, CC2D1A also functions as a key innate immune adaptor. It bridges RIG-I-like receptors to the mitochondrial adaptor IPS-1, facilitating type I interferon induction in response to RNA virus infection [14], and enhances NF-κB transcriptional activation via the canonical IKK pathway [4,9]. In addition, a recent study further showed that CC2D1A exploits endosomes to mediate RIG-I signaling and promote antiviral responses [15]. While these biochemical functions have been independently characterized, how these domains are structurally arranged and functionally coordinated remains poorly understood.

Recent studies suggest that these domains act not independently but rather as a cooperative regulatory module. For example, repression of 5-HT1A and D2 receptor genes depends on both the coiled-coil region and C2 domains [2], while lipid binding and Akt activation require the concerted function of the fourth DM14 (DM14-4) and C2 [3,5]. The functional interplay among these domains suggests the existence of a higher-order modular structure critical for integrating nuclear and cytoplasmic roles of CC2D1A [5].

This hypothesis is particularly relevant in light of clinical findings. The most common truncation mutation associated with NSID eliminates a contiguous DM14-4-CC-C2 segment [8], leading to a loss of transcriptional repression, phospholipid interaction, and NF-κB activation [5]. Despite this genetic and functional relevance, the molecular mechanism by which these three domain units support CC2D1A function and how its absence leads to cognitive and behavioral deficits remains unresolved.

To date, no atomic resolution structural data have been reported for any region of human CC2D1A. Two structures from its *Drosophila* homolog Lgd have been solved: the third DM14 domain (PDB: 5VNY) and its complex with CHMP4B (PDB: 5VO5), revealing a hairpin-like α-helical fold that interacts electrostatically with endosomal sorting complex required for transport-III (ESCRT-III) components [6,16]. In addition, the coiled-coil and C2 domains of the *Drosophila* CC2D1B ortholog have been structurally resolved (PDB: 6EI6) [17], offering partial insight into the modular organization of CC2D1-family proteins. However, there is no structural information available for any corresponding region of human CC2D1A. Most importantly, none of the existing models capture the integrated architecture of the DM14-CC-C2 three domains module, which is central to the transcriptional repression and signaling functions of CC2D1A and frequently disrupted in disease-associated truncation mutants.

Here, we report the first crystal structure of the human CC2D1A DM14-4-CC-C2 domain module. This structure provides atomic-resolution insight into a disease-relevant multi-domain unit, reveals a conserved basic surface suitable for DNA recognition, and offers a mechanistic basis for understanding how CC2D1A functions as a transcriptional and signaling integrator.

## Methods

### DNA construction, protein expression, and purification

The DNA fragments encoding human CC2D1A_137–402_ and CC2D1A_491–__810_ were cloned into the pSol-His vector of the Lucigen Expresso solubility and expression screening system using the TAKARA In-Fusion cloning kit. The resulting construct encodes an N-terminal His_6_-tagged protein and was transformed into *Escherichia coli* BL21(DE3) for protein expression. Transformed cells were cultured in LB medium containing 30 μg/ml kanamycin at 37°C until the optical density at 600 nm (OD_600_) reached 0.6. Cultures were then cooled on ice for 30 min, followed by induction with 0.2% final concentration of L-rhamnose at 18°C overnight. Cells were harvested by centrifugation at 5000 × ***g*** for 20 min at 4°C and resuspended in lysis buffer (20 mM Tris–HCl pH 8.0, 500 mM NaCl, 5 mM imidazole). Cell lysis was performed using a microfluidizer (Microfluidics), and lysates were clarified by centrifugation at 30 000 × ***g*** for 50 min at 4°C. The supernatant was filtered through a 0.22 μm membrane and applied to a 5 ml HisTrap HP column (Cytiva) pre-equilibrated with lysis buffer. After washing with 10 column volumes of buffer containing 50 mM imidazole, bound proteins were eluted with 200 mM imidazole in the same buffer. Eluted proteins were dialyzed against 20 mM Tris–HCl (pH 8.0), 100 mM NaCl, and further purified by size-exclusion chromatography on a Superdex 200 16/60 GL column (GE Healthcare) using an ÄKTA Pure system, equilibrated with 20 mM Tris–HCl (pH 8.0), 100 mM NaCl, and 2 mM β-mercaptoethanol. Peak fractions were analyzed by SDS–PAGE (8%–12% acrylamide), and the target protein was concentrated to ∼5 mg/ml and stored at −80°C.

### Analytical ultracentrifugation

Analytical ultracentrifugation (AUC) experiments were performed using a Beckman Coulter XL-A analytical ultracentrifuge equipped with a Ti An-60 rotor. Sedimentation velocity analysis was conducted to characterize the oligomeric states and molecular weights of CC2D1A constructs: CC2D1A_137–__402_ and CC2D1A_491–__810_. Prior to centrifugation, protein samples were purified by size-exclusion chromatography and dialyzed into buffer containing 20 mM Tris–HCl (pH 8.0) and 100 mM NaCl. The density and viscosity of the buffer were calculated using the *SEDNTERP* program [18], based on its composition. Absorbance at 250 nm was monitored in real-time. Data were analyzed using *SEDPHAT* (http://analyticalultracentrifugation.com). Sedimentation equilibrium experiments were conducted at rotor speeds of 3200, 3800, and 7000 rpm at 4°C. Absorbance at 250 nm was recorded at each speed after equilibrium was reached, typically within 24–48 h. The resulting radial concentration profiles were globally fitted using *SEDPHAT*.

### Electromobility shift assay

Double-stranded DNA substrates were prepared by annealing a 5′ cyanine-3 (Cy3)-labeled 24-nt (5′-TGAGTGGGATAAGCAAGCCCTTCG-3′), containing the D2-DRE sequence, with its complementary strand to form a duplex DNA [2]. Band-shift assays were performed in a total volume of 20 μl containing 20 mM Tris–HCl (pH 8.0), 100 mM NaCl, 20 nM duplex DNA, and varying concentrations of CC2D1A protein. The reaction mixtures were incubated at room temperature for 30 min. Following incubation, native gel loading dye (final concentration: 10 mM Tris–HCl pH 7.6, 5% glycerol) was added directly to each sample. Samples were resolved by electrophoresis on 8% TBE polyacrylamide gels prepared in 0.5× TBE buffer (45 mM Tris-borate pH 8.0, 1 mM EDTA) at 70 V for 100 min. Gels were scanned immediately after electrophoresis using the Cy3 channel on a Typhoon FLA 9000 imaging system (GE Healthcare) to detect fluorescence-labeled DNA bands.

### Fluorescence polarization assay

Fluorescence polarization (FP) binding assays were conducted to assess the equilibrium DNA-binding affinity of various CC2D1A constructs. DNA substrates were labeled with Cy3 at the 5′ end, allowing detection of FP changes upon protein binding. A two-fold serial dilution series of CC2D1A proteins, starting from 500 μM, was prepared in storage buffer (20 mM Tris–HCl pH 8.0, 100 mM NaCl). Each dilution was pre-incubated with 5 nM Cy3-labeled DNA at room temperature. The FP signal of the Cy3-labeled DNA in buffer alone served as the baseline for the unbound state. Changes in FP upon protein binding were measured using a Paradigm plate reader (Molecular Devices), with excitation at 535 nm and emission detection at 595 nm. Binding curves were generated from three independent experiments, and the apparent dissociation constant (Kd) was calculated as the protein concentration required for half-maximal binding. Error bars represent the standard deviation from three independent experiments (*N* = 3).

### Protein crystallization and data collection

Crystallization trials used 5 mg/ml CC2D1A_491–810_ in 20 mM Tris–HCl pH 8.0, 100 mM NaCl, and β-mercaptoethanol. Initial screening was performed using a Phoenix robot platform (Rigaku) with commercially available screen reagents (Hampton Research, Molecular Dimension, Rigaku, and Qiagen Ltd) and at 20°C. Crystals (parallelogram-shaped plate crystals) that diffracted to ∼4.5 Å resolution were obtained for the (0.04 M citric acid, 0.06 M Bis-Tris propane pH 6.4), 20% w/v polyethylene glycol 3350 trial. Furthermore, crystals suitable for X-ray diffraction were obtained by modifying the pH value to 6.0, decreasing polyethylene glycol 3350 concentration to 16% w/v, and adding 5% glycerol by the hanging-drop-vapor diffusion method. The diffraction data of CC2D1A_491-810_ crystals were generated using synchrotron X-ray radiation at TPS 05A Beamline of the National Synchrotron Radiation Research Center, Taiwan, and collected at a Rayonix MX300HS CCD Area Detector (Rayonix, LLC) at 100 K.

### Structure determination and refinement

X-ray diffraction dataset for CC2D1A_491–810_ structure was processed using *DENZO* and *SCALEPACK* in HKL2000 [19]. The phase of the CC2D1A_491–810_ structure was determined by molecular replacement (MR) in *Phaser* [20]. The search model was generated by the Phyre^2^ server [21] using the C2 domain of *Drosophila* Lgd (PDB ID: 6EI6) [17]. The C2 domain part exhibited a density map, however, the N-terminal part lacked clear density. Therefore, we used the autobuild from the *PHENIX* package to build the model and refinement. The initial phase of CC2D1A_491–810_ was improved by manual model building (*Coot*) [22] combined with MR (*PHENIX*) and further improved to a 2.3 Å resolution by using the maximum likelihood density modification algorithm in *PHENIX*. The native structure was energy minimized and then annealed by *PHENIX* and *CCP4* [20,23]. By using the water-picking routine in *PHENIX*, 126 water molecules were found in the structure. The final model has an *R* factor of 21.8% to reflections >2σ, between 30.0 and 2.3 Å resolutions, and an *R*_free_ value of 27.4% for 5% of randomly chosen reflections. A *PHENIX*-generated Ramachandran plot for the CC2D1A_491–810_ angles indicates that 96% are in the most favored regions and that 3.9% are in allowed regions. *PyMol* was used to generate the Figures [24]. Detailed X-ray diffraction data and structural refinement statistics are summarized in Supplementary Table S1.

### Cross-linking of CC2D1A_491–810_ using disuccinimidyl suberate cross-linker

Cross-linking reactions were performed in a total volume of 100 μl, containing CC2D1A_491–__810_ at an initial concentration of 10 μM followed by a two-fold serial dilution. Each protein sample was incubated with 0.5 mM disuccinimidyl suberate (DSS) (Thermo Scientific™) in 1× PBS buffer pH 7.4 (137 mM NaCl, 2.7 mM KCl, 8 mM Na_2_HPO_4_, and 2 mM KH_2_PO_4_) at room temperature for 30 min. Reactions were quenched by adding 5 μl of 1 M Tris–HCl (pH 8.0) to a final concentration of 50 mM. Cross-linked products were resolved on 4%-12% SDS–PAGE gels and visualized by Coomassie Brilliant Blue staining.

## Results

### Characterization and DNA-binding properties of CC2D1A truncation constructs

**Figure 1:**
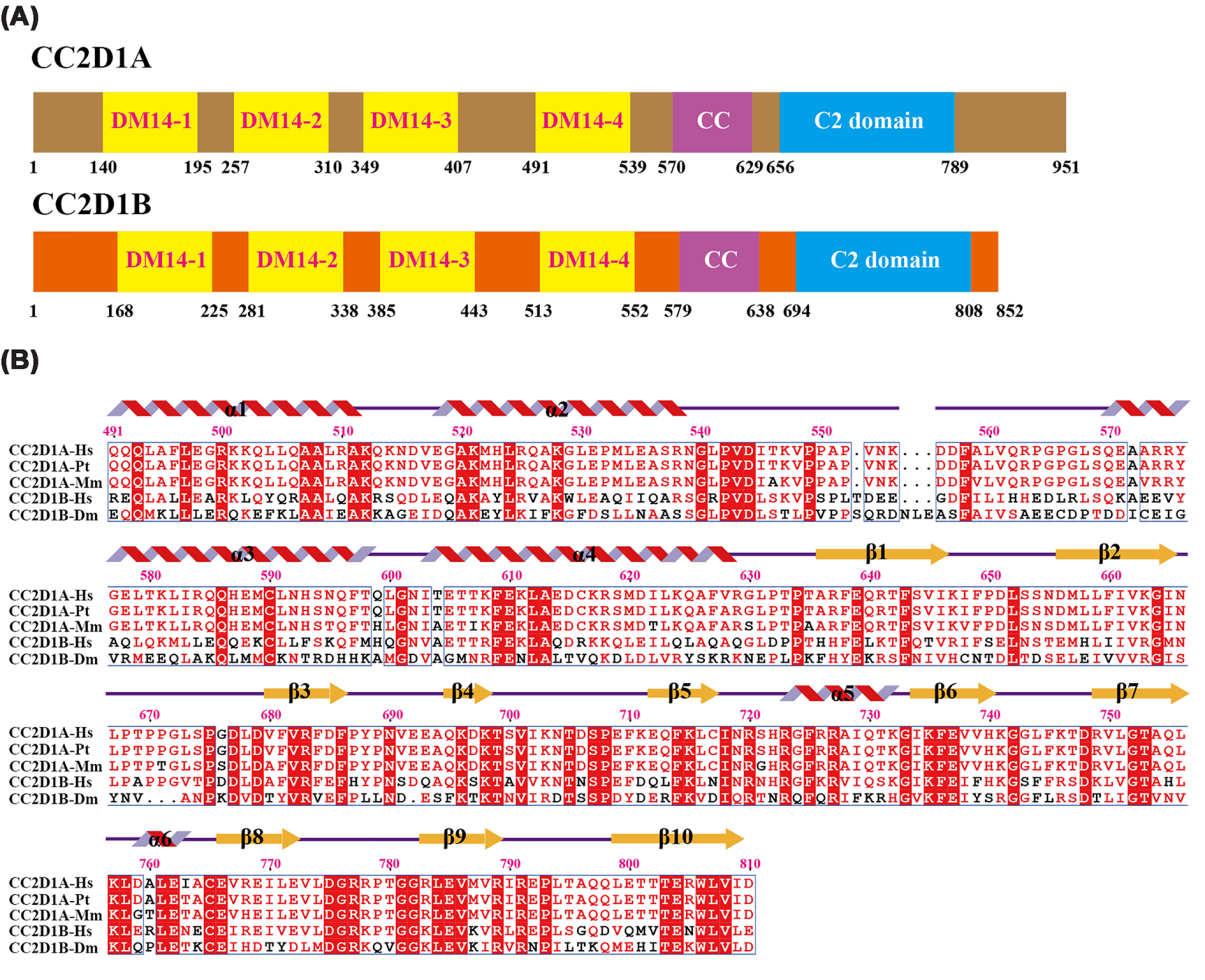
Domain organization and sequence conservation of CC2D1 family proteins across species (**A**) Schematic domain architecture of human CC2D1A and *Drosophila melanogaster* CC2D1B (Lgd), showing four DM14 domains, a coiled-coil (CC) region, and a C-terminal C2 domain. (**B**) Multiple sequence alignment of human CC2D1A_491–__810_ and its homologs from *Homo sapiens* (Hs), *Pan troglodytes* (Pt), *Mus musculus* (Mm), and *Drosophila melanogaster* (Dm). Secondary structure elements derived from the CC2D1A_491–__810_ crystal structure are annotated above the alignment. Strictly conserved residues are boxed in red; residues conserved in at least two of the four sequences are highlighted in blue boxes. Sequence alignments were performed using Clustal Omega and visualized with ESPript3 [45].

**Figure 2:**
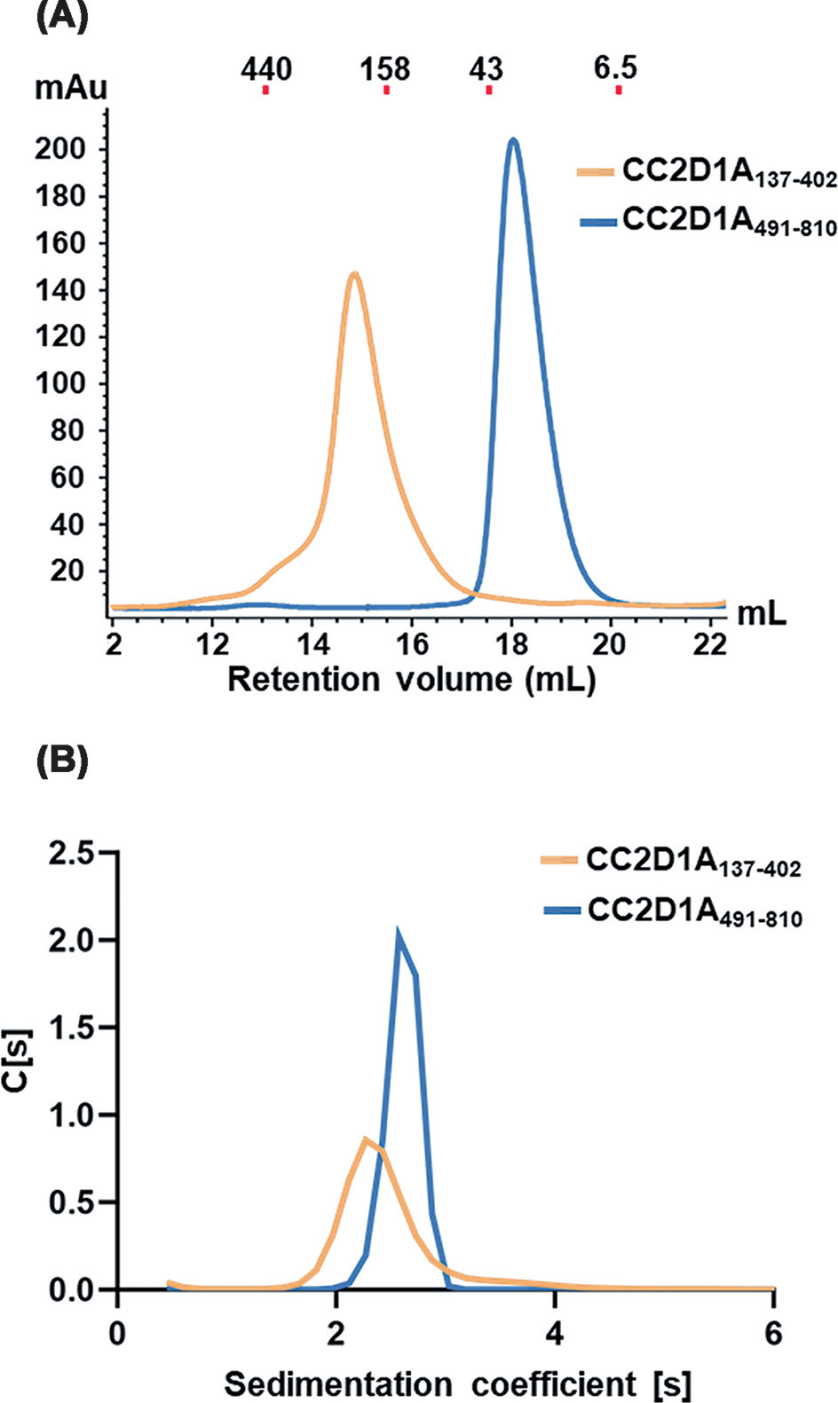
Oligomeric state analysis of CC2D1A constructs by size-exclusion chromatography and sedimentation velocity AUC **(A**) Gel filtration elution profiles of purified CC2D1A_137–__402_ (orange) and CC2D1A_491–__810_ (blue) on a Superdex 200 10/300 column. Molecular weight standards are indicated at the top. (**B**) Sedimentation velocity profiles of the same CC2D1A constructs, analyzed using *SEDPHAT*.

Human CC2D1A is a 951-residue protein composed of four DM14 domains, a central coiled-coil region, and a C-terminal C2 domain (Figure 1A). Although CC2D1A has been extensively studied in the context of transcriptional repression and intracellular signaling, structural information has remained elusive. First, we attempted to obtain the full-length protein and various fragment constructs. However, the full-length protein and most constructs exhibited severe degradation or insolubility upon purification. We successfully purified two CC2D1A constructs: CC2D1A_137–402_ (DM14-1 to DM14-3) and CC2D1A_491–810_ (DM14-4, coiled-coil, and C2 domains). Both constructs were subjected to size-exclusion chromatography, which revealed monodisperse elution profiles (Figure 2A). However, the two constructs exhibited different elution behaviors relative to their theoretical molecular weights. CC2D1A_491–810_ (36 kDa) eluted at a position consistent with its expected size, while CC2D1A_137–402_ (29 kDa) eluted earlier than expected. This difference suggests that the N-terminal construct (CC2D1A_137–402_) may adopt a more extended or non-globular conformation in solution. To further characterize the solution behavior of each construct, we performed sedimentation velocity AUC on the gel filtration-purified fractions. The sedimentation coefficient distributions c(s) were 2.1 S for CC2D1A_137–402_ and 2.7 S for CC2D1A_491–810_ (Figure 2B). In addition, the corresponding molecular weights estimated from the sedimentation data are 29 kDa and 36 kDa, respectively. These results are consistent with their theoretical values, confirming that both fragments exist as monomers in solution.

**Figure 3:**
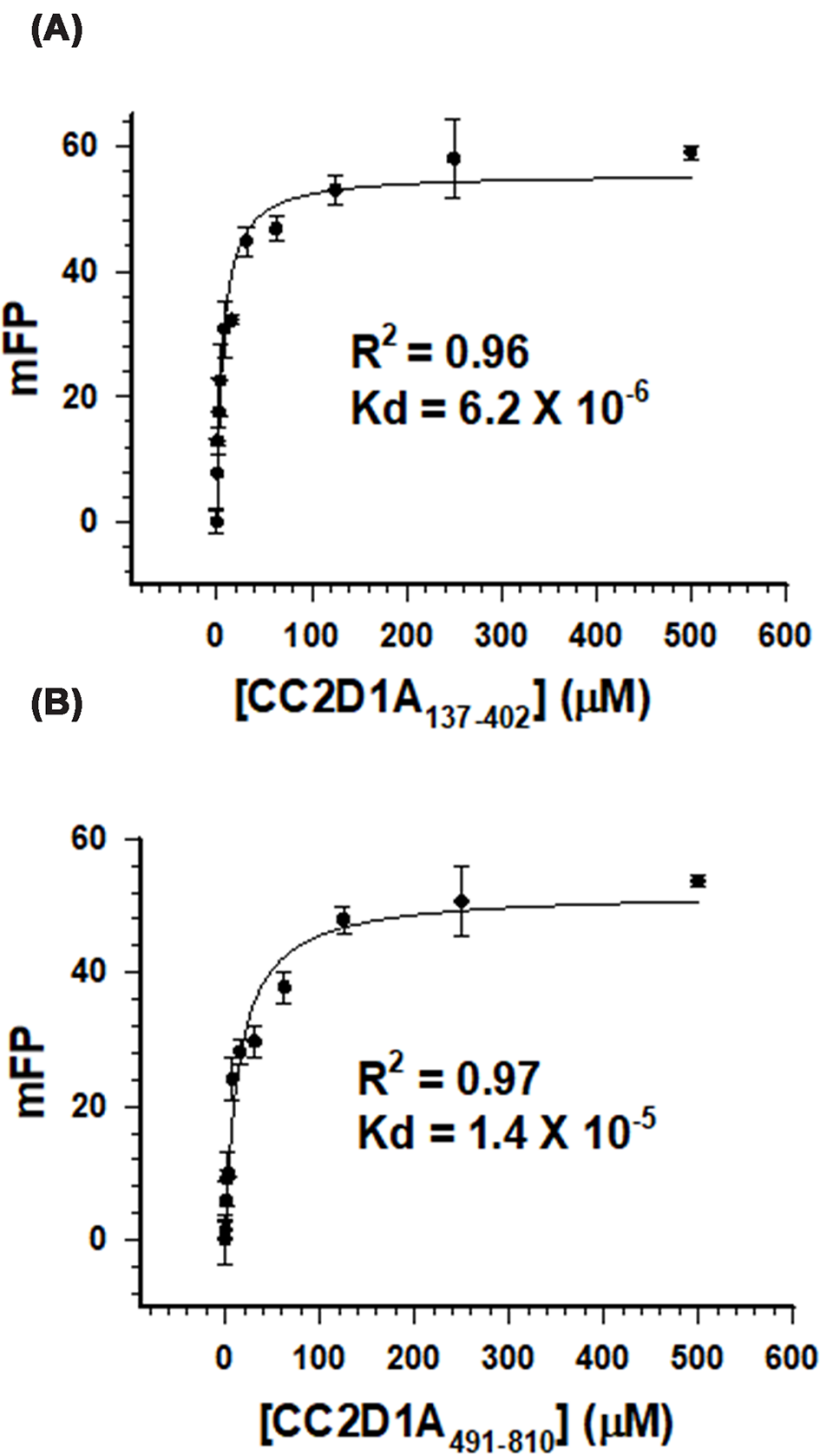
DNA-binding ability of CC2D1A constructs, as determined by FP binding isotherms Cy3-labeled double-stranded DNA was incubated with (**A**) CC2D1A_137–402_ and (**B**) CC2D1A_491–810_ at increasing protein concentrations. All measurements were performed in triplicate (*N* = 3), and error bars represent the standard deviation of the mean. Solid lines indicate the fitted binding curves using a one-site saturation model.

Previous genetic and biochemical studies have established that CC2D1A functions as a transcriptional repressor of both the serotonin-1A (5-HT1A) and dopamine D2 receptor (DRD2) genes by binding to conserved DRE elements, including a highly conserved sequence in the second intron of the DRD2 gene (D2-DRE) [25–27]. Notably, partial deletion of the C2 domain impairs repression of the 5-HT1A promoter while retaining weak DRE-binding activity, suggesting that multiple regions within CC2D1A may cooperate to mediate DNA binding [1]. To assess the DNA-binding abilities of these two constructs, we designed a 24-bp sticky-end DNA probe (5′-TGAGTGGGATAAGCAAGCCCTTCG-3′) and performed electrophoretic mobility shift assays (EMSA). EMSA results revealed that all three constructs bound the DNA probe in a concentration-dependent manner (Supplementary Figure S1). FP assays were performed to quantify the DNA-binding affinities of each construct. The measured dissociation constants (Kd) for CC2D1A_137–402_ and CC2D1A_491–810_ were 6.2 μM and 14 μM, respectively (Figure 3). Interestingly, CC2D1A_137–402_, containing DM14-1 to DM14-3, displayed the highest affinity, approximately two-fold stronger than CC2D1A_491–810_, which contains the C-terminal DM14-4, coiled-coil, and C2 domains, suggesting that the N-terminal DM14 repeats are the primary determinants of DNA interaction. The comparable DNA-binding affinities suggest that CC2D1A may recognize nucleic acid structures in a sequence-independent manner and might possibly facilitate scaffold-like functions.

### Overall domain architecture and tertiary arrangement of CC2D1A_491–810_

**Figure 4:**
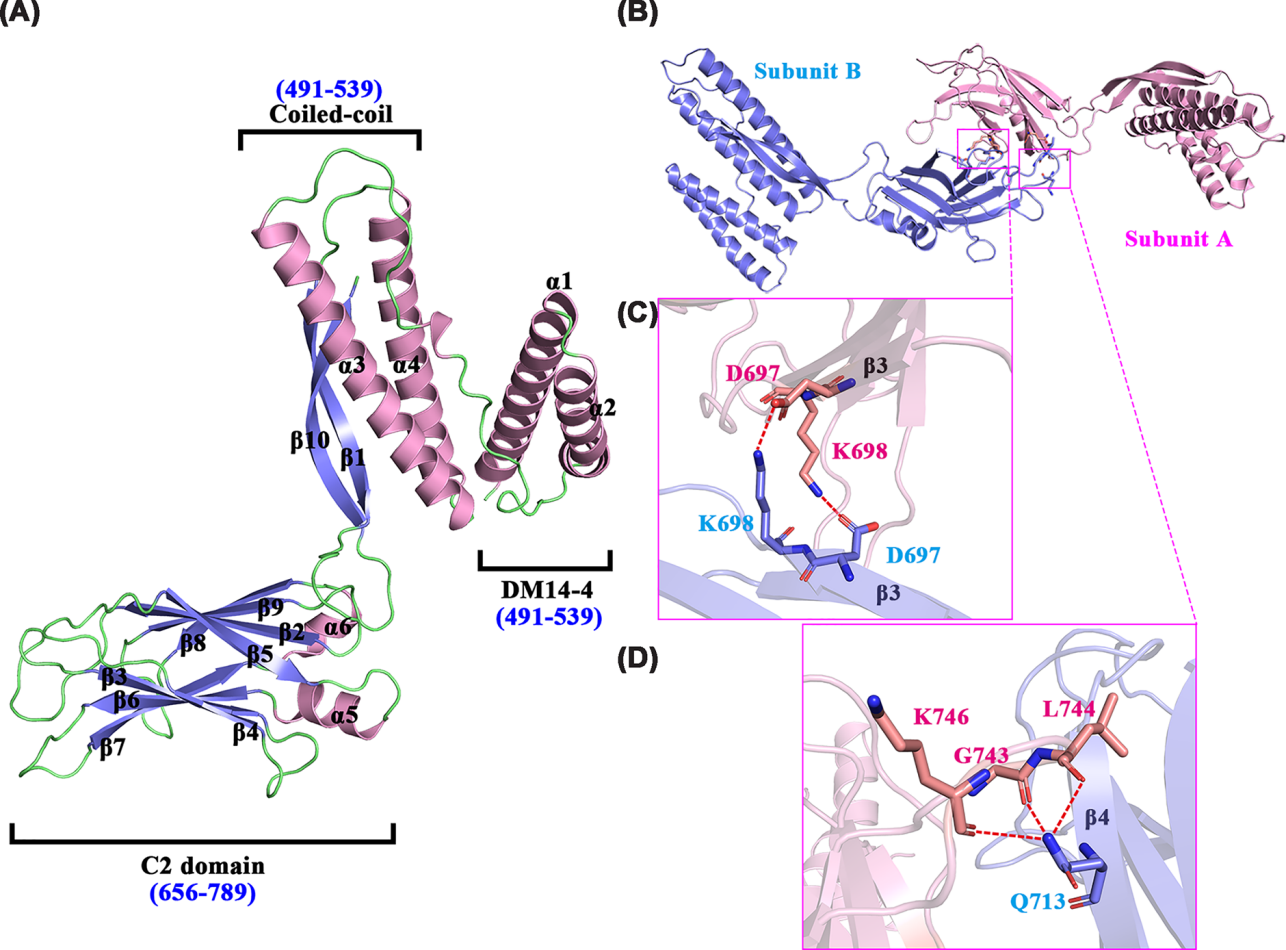
Structural architecture and dimer interface of CC2D1A_491__–__810_ (**A**) The CC2D1A_491–__810_ monomer is shown as a cartoon, and the DM14-4 domain (residues 491–539), the coiled-coil region (residues 570–629), and the C-terminal C2 domain (residues 656–789) are labeled. Secondary structure elements are labeled and color-coded: α-helices in pink, β-strands in blue, and loops in green. (**B**) The CC2D1A_491–__810_ homodimer, showing subunit A (pink) and subunit B (blue). Dimerization is mediated by reciprocal interactions between the β3 strands of each C2 domain. (**C**) Close-up view of the β3–β3 interface, showing two inter-subunit salt bridges formed between Asp697 and Lys698 of opposing monomers. (**D**) Additional hydrogen bonding interactions at the dimer interface, where Gln713 from Subunit B forms three hydrogen bonds with Leu744, Gly743, and Lys746 from Subunit A, contributing to interface stability.

Despite extensive efforts to crystallize these two constructs and their DNA complexes, we were only able to obtain crystals and solve the structure for CC2D1A_491–810_ (containing DM14-4, coiled-coil, and C2 domains). The crystal structure of this fragment, encompassing residues 491–810, at 2.3 Å resolution. As shown in Figure 4, the structure reveals a dimer conformation, and each monomer assembles into a compact three-domain architecture consisting of the fourth DM14 domain (DM14-4; Gln491–Asn539), a coiled-coil region (Gln570–Arg629), and a C-terminal C2 domain (Asp656–Arg789). DM14-4 adopts an α-helical hairpin fold comprising α1 and α2, connected by a long flexible loop (Gly540–Ser569) to a ∼50 Å coiled-coil segment formed by α3 and α4 (Figure 4A). This long linker loop (Gly540–Ser569) interacts extensively with both DM14-4 and the coiled-coil helices through hydrophobic and hydrogen bonding interactions, likely stabilizing the overall fold (Figure 4A).

Similar to previously reported C2 domain structures [17], the C-terminal C2 domain of CC2D1A_491–810_ adopts a typical β-sandwich fold composed of eight antiparallel β-strands, consistent with a type-II C2 domain topology (Figure 4A and Supplementary Figure S2). Notably, two short α-helices (α5 and α6) are observed in the loops between β5–β6 and β7–β8 (Figure 4A). These helices appear to be a characteristic feature unique to the CC2D1 protein family. A distinctive structural hallmark of CC2D1A_491–810_ is the terminal antiparallel β-sheet formed between β1 (Ala636–Ile646) and β10 (Leu799–Ile809). This β-sheet packs tightly against the coiled-coil helices, burying a substantial portion of the coiled-coil surface and shielding it from solvent exposure, which suggests a role in structural stabilization. The β1–β10 interface thus appears to function as a structural anchor, integrating DM14-4, the coiled-coil region, and the C2 domain into a cohesive and compact modular assembly (Figure 4A).

### Antiparallel β1–β10 sheet anchors domain packing and reinforces structural integrity

**Figure 5:**
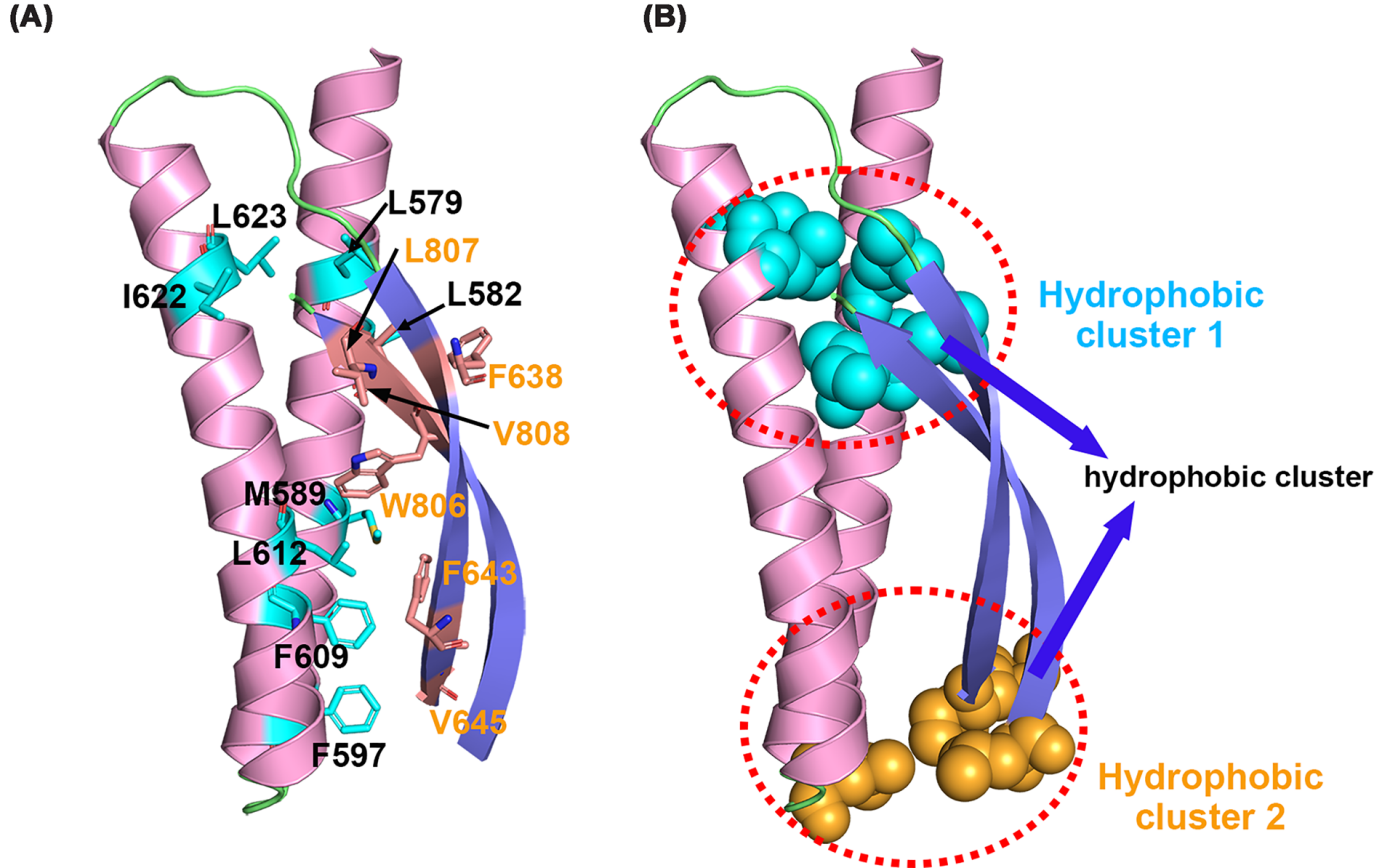
Hydrophobic interactions between β-strands and the coiled-coil region stabilize the CC2D1A modular core (**A**) Hydrophobic residues at the interface between the β1–β10 sheet and the coiled-coil helices are shown. Residues from the coiled-coil (pink) are labeled in cyan and residues from the β-sheet (blue) in orange. (**B**) Surface representation of the same region and orientation showing two spatially segregated hydrophobic clusters (hydrophobic cluster 1 in cyan and cluster 2 in orange), which mediate packing between the β-sheet and the coiled-coil helices.

Although β1 and β10 are far away in the primary sequence (Figure 1B), they fold together in the structure of CC2D1A_491–810_ to form a contiguous antiparallel β-sheet spanning approximately 30 Å (Figure 4A). This β1–β10 sheet inserts into the core interface between the coiled-coil and C2 domains. Its positioning suggests a potential role in mediating domain–domain packing and stabilizing the overall architecture. (Figures 4A and 5). At the bottom of one side, this β1-β10 sheet forms salt bridges with residues in the C2 domain (Lys647 and Glu800), stabilizing a defined but flexible inter-domain geometry (subunit A: ∼90° and subunit B: ∼110°) with the C2 domain (Supplementary Figure S3). On the other side, the coiled-coil region and β1–β10 sheet contain many hydrophobic residues (Figure 5A). The β-sheet establishes extensive hydrophobic interactions with the coiled-coil region, effectively embedding itself into the tertiary architecture (Figure 5A). This β-sheet establishes extensive hydrophobic contacts with both flanking regions, engaging two major clusters of nonpolar residues identified via ProteinTools analysis (Figure 5B) [9]. Hydrophobic cluster 1, comprising residues such as Leu560, Ile583, and Leu623, stabilizes the N-terminal half of the sheet, while cluster 2, centered around Leu600, Ile646, and Leu799, secures its C-terminal end. This dual interface suggests that the antiparallel β-sheet plays a key structural role in bridging domain boundaries. Together, these interactions imply that the β1–β10 sheet may function as an internal stabilizer that modulates the overall compactness of the CC2D1A three-domain module. Its strategic position and interfacial interactions likely contribute to maintaining the conformational integrity required for functional modular coordination.

### The DM14-4-CC-C2 module forms a structurally integrated unit with C2-driven dimerization

The AUC and gel filtration analyses consistently demonstrate that CC2D1A_491–__810_ exists as a monomer in solution (Figure 2), however, the crystal structure reveals a dimeric assembly mediated by the C2 domain (Figure 4B,C). Within the asymmetric unit, two subunits adopt distinct conformations, while subunit-A displays a nearly orthogonal (∼90°) arrangement between its coiled-coil and C2 regions. In addition, Subunit-B bends outward, forming a ∼110° angle, and two subunits with a ∼20° deviation between the two molecules (Supplementary Figure S3A). To determine the most likely interaction interface that could represent a major structural element in dimer formation, we used the PISA server [28] to measure the surface areas of dimer interface. The buried surface within dimer is 502.4 Å^2^ (Figure 4B), and the interaction between the two monomers contributes to the assembly and stabilization of the dimer. The dimerization is centered at the C2 domains, where reciprocal β3–β3 interactions mediate interface formation. This interface is stabilized by two inter-subunit salt bridges formed between Asp697 and Lys698, as well as a hydrogen bond network in which Gln713 from one subunit engages Leu744, Gly743, and Lys746 of the opposing subunit (Figure 4B–D). Moreover, these interfacial residues are highly conserved among CC2D1A homologs (Figure 1B). This structural observation contrasts with prior assumptions, as the C2 domain has not previously been implicated in mediating dimerization. Notably, similar C2-mediated dimerization has been observed in other proteins, including PI3K-C2α, reinforcing the plausibility of this interaction mode [29].

To investigate the possibility that CC2D1A undergoes concentration-dependent dimerization mediated by its C2 domain, we performed a DSS cross-linking assay. Consistent with our AUC and gel filtration results (Figure 2), CC2D1A_491–810_ predominantly exists as a monomer in solution. However, upon increasing protein concentration, we observed a progressive accumulation of dimeric species (Supplementary Figure S4), supporting the notion that dimerization is concentration-sensitive. These results are in agreement with the crystal structure, which reveals a dimeric assembly stabilized by C2 domain-centered interactions (Figure 4B–D). Notably, this structural mode involves reciprocal β3–β3 pairing and a conserved inter-subunit network of salt bridges and hydrogen bonds, indicating a propensity for dimerization. Although we cannot rule out additional cellular factors, such as metal ions, lipid membranes, or binding partners that may further regulate or stabilize this dimeric state, our findings provide biochemical evidence that CC2D1A can dimerize under certain conditions. This raises the possibility that C2-mediated dimerization may serve as a regulated feature *in vivo*, potentially contributing to its scaffolding or signaling roles. Further studies are warranted to delineate the physiological triggers and functional consequences of this dimeric configuration.

### Structural conservation and potential DNA-binding capability of the DM14 domains

**Figure 6:**
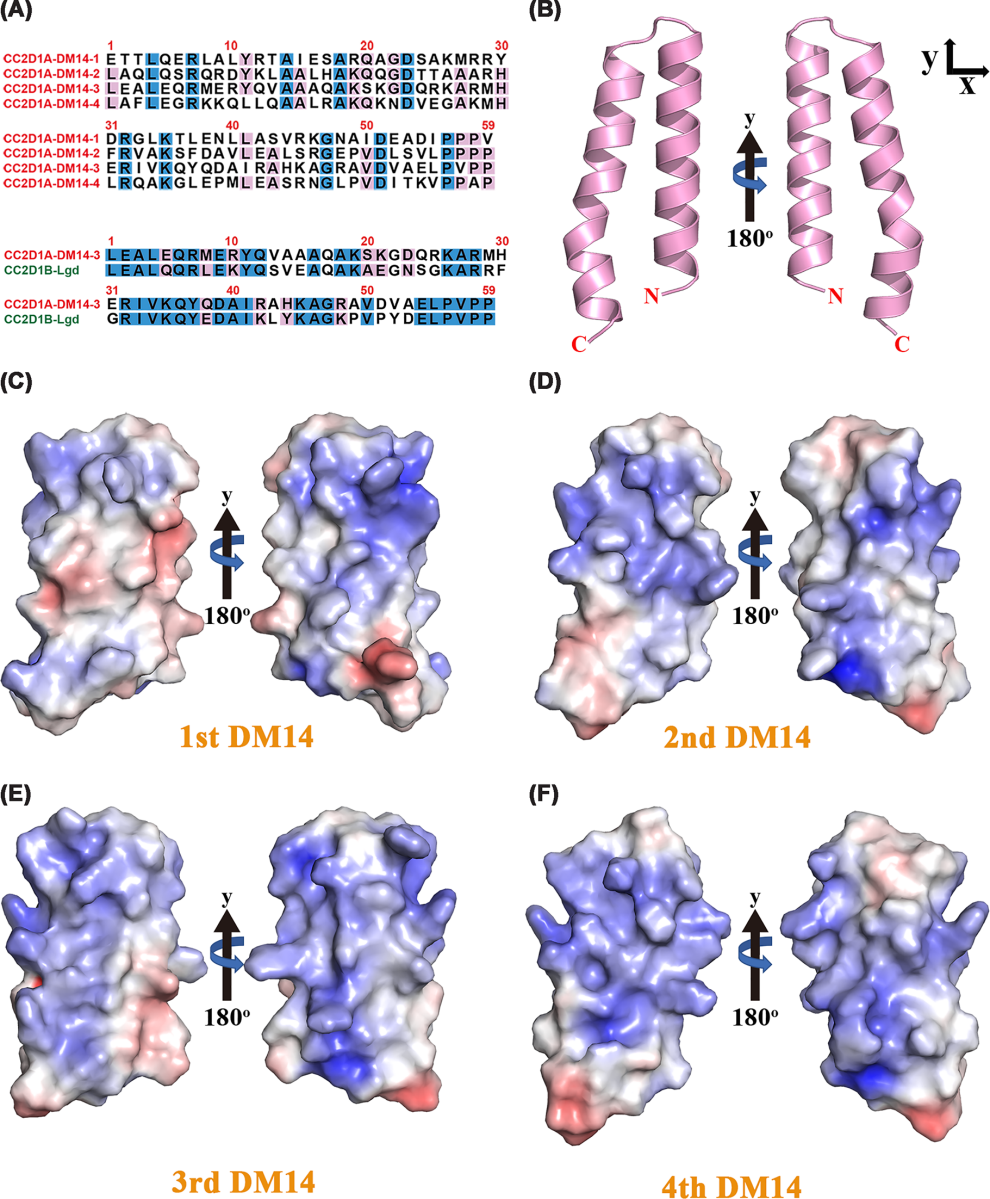
Sequence and electrostatic features of the DM14 domains in CC2D1A (**A**) Pairwise sequence alignment of all four DM14 domains from human (top), and sequence alignment of DM14-3 domains from human CC2D1A and *Drosophila melanogaster* Lgd (bottom). (**B**) Ribbon diagram of the DM14-4 domain from the CC2D1A_491–__810_ crystal structure. Electrostatic surface representations of the four DM14 domains. (**C**) DM14-1, (**D**) DM14-2, and (**E**) DM14-3 were modeled using AlphaFold2; (**F**) DM14-4 was derived from the crystal structure. Electrostatic potential maps were generated using the APBS plugin in PyMOL, with red and blue indicating negative and positive surface charges, respectively.

The DM14 domains of CC2D1A represent a family-specific motif whose precise molecular function remains only partially understood. The DM14 domain, characterized by a typical hairpin conformation, has been implicated in protein–protein interactions [6]. Previous studies of CC2D1A have demonstrated that the first DM14 is essential for PDE4D binding; however, deletion of DM14-1 to -3 abolishes complex translocation and leads to constitutive PDE4D S126 phosphorylation through PKA [30]. The third DM14 of CC2D1A interacts with CHMP4B, a protein involved in ESCRT-III, and CC2D1A acts as a negative regulator of CHMP4B function [31]. In addition, the fourth DM14 domain impairs CC2D1A-mediated Akt activation by disrupting PDK1 interaction and PIP3 binding, underscoring its importance in signal transduction [3]. Although the four DM14 domains exhibit structural redundancy, they also show partial functional complementarity, as removal of one or two DM14s leads to only partial reduction in activity [4,5]. However, sequence alignment of the four DM14 domains revealed that the overall sequence identity was low (∼31.2%), while the identity between DM14-2 and DM14-4 was relatively high (44.2%) (Figure 6A and Supplementary Table S2). Notably, CC2D1A DM14-3 exhibited high sequence similarity with the third DM14 domain of *Drosophila* Lgd (Figure 6A), suggesting an evolutionarily conserved role for this repeat across species.

Despite their low sequence identity, we investigated whether the DM14 domains of CC2D1A share structural similarity that enables them to complement each other functionally. To explore structural conservation among DM14 domains, we modeled the structures of DM14-1, DM14-2, and DM14-3 using AlphaFold2 [32] and compared their electrostatic surface potentials with that of DM14-4 derived from our crystal structure. All surface representations were visualized following the same orientation shown in Figure 6B to facilitate direct comparison (Figure 6C-F). The electrostatic surface mapping of the DM14 domains revealed a positively charged patch on each DM14 domain (Figure 6C-F). This positively charged surface patch is created by a cluster of conserved basic residues (Figure 6). The presence of this conserved positive surface prompted us to investigate whether the DM14 domains could bind DNA directly. These conserved surface patches likely serve as generalized scaffolds for macromolecular interactions, providing the structural foundation that enables DM14 repeats to function in interchangeable yet cooperative roles in protein interactions and cellular signaling. Notably, the sequence divergence among DM14 domains may underpin their functional specialization.

### C2 domain of CC2D1A lacking calcium-binding features

Similar to previously reported C2 domain structures [33,34], the human CC2D1A C2 domain exhibits a typical β-sandwich with eight antiparallel β-strands. The topology of the eight β-strand arrangement indicates a type-II C2 domain (Supplementary Figure S2). Two short α-helices (α5 and α6) in the C2 domain (Figures 1B and 4A), which are characteristic features unique to the CC2D1 family, were also observed in human CC2D1A. These helices are located in the loops connecting β5 to β6 and β7 to β8 strands, respectively (Figure 4A).

Although C2 domains are often implicated in Ca^2+^-dependent lipid binding, previous studies have suggested that the C2 domain of CC2D1A does not function in this manner due to the lack of five conserved aspartate residues required for canonical calcium coordination. Subsequent biophysical analysis confirmed the absence of calcium-binding activity *in vitro* [4,9]. To further investigate, we compared the CC2D1A C2 domain with the Ca^2+^-binding type II C2 domain of phospholipase C-δ1 (PLCδ1; PDB: 1DJI) [35] and examined the canonical Ca^2+^-binding regions (CBR1–CBR3). Interestingly, closer inspection of our structure revealed that several aspartate residues, including Asp677, Asp679, Asp706, and Asp748, are present within CBR1–CBR3 (Supplementary Figure S5). These residues are conserved across the CC2D1A and CC2D1B protein family (Figure 1B), suggesting evolutionary retention of negative surface charge in this region. Although acidic residues are present in both proteins, the spatial configuration of CBR1, CBR2, and CBR3 in CC2D1A is considerably more expanded than the compact Ca^2+^-coordinating geometry observed in PLCδ1 (Asp653, Asp706, Asp708, and Asp714 in PLCδ1). This expanded geometry likely precludes effective Ca^2+^ coordination, consistent with the absence of bound ions in the electron density map and biophysical data indicating a lack of calcium-binding activity [9].

These features strongly suggest that the CC2D1A C2 domain functions in a calcium-independent manner. Its role may instead involve membrane association or recruitment of signaling proteins via electrostatic or hydrophobic interactions, consistent with its previously reported importance in NF-κB activation [4].

### Comparative structural features between human CC2D1A and drosophila lgd

To date, structural information for the CC2D1 protein family is available only from the *Drosophila* homolog Lgd, a CC2D1B ortholog. Two structures have been reported: one comprising the third DM14 domain alone (PDB ID: 5VNY) [6] and the other encompassing the coiled-coil region together with the C2 domain (PDB ID: 6EI6) [17]. Previous studies demonstrated that the third DM14 domain of Lgd regulates the activity of Shrub, a component of the ESCRT-III. Specifically, the third DM14 domain alone is sufficient to mask the homopolymerization interface of Shrub [6].

Among the four DM14 domains in Lgd, substantial sequence divergence has been observed: DM14-1 and DM14-3 are more similar to each other than to DM14-2 or DM14-4, whereas DM14-2 and DM14-4 are likewise more closely related to each other [36]. In contrast, sequence alignment of the four DM14 domains in human CC2D1A revealed greater overall conservation, with approximately 31.2% sequence identity among them (Supplementary Table S2). Notably, a relatively higher identity (44.2%) was observed between DM14-2 and DM14-4. Furthermore, sequence alignment between the third DM14 domains of Lgd and human CC2D1A showed 81.6% similarity (Figure 6B), indicating strong evolutionary conservation across species.

Structural analysis further revealed that the third DM14 domain of Lgd possesses a conserved positively charged surface patch formed by a cluster of basic residues, including Lys387, Arg389, Arg390, and Arg393 [6]. Similarly, the third DM14 domain of human CC2D1A retains these basic residues with high conservation, whereas the fourth DM14 domain conserves all but one (Figure 6). These findings suggest that the DM14 domains in human CC2D1A may exhibit greater complementarity and structural preservation compared with those in Lgd, potentially enhancing electrostatic interactions akin to those observed in the Lgd/Shrub complex.

**Figure 7:**
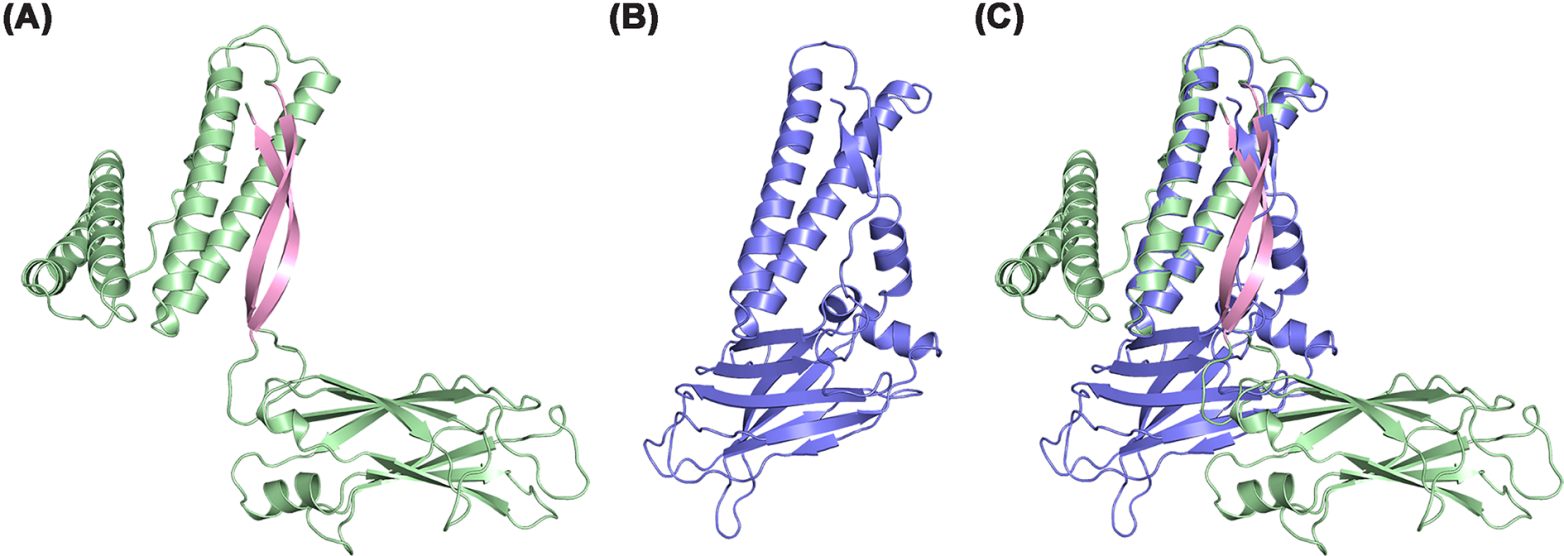
Structural comparison of the CC2D1A_491__–__810_ with the Lgd coiled-coil-C2 region (**A**) Crystal structure of a CC2D1A_491–__810_ monomer, highlighting the antiparallel β-sheet (pink) formed between the coiled-coil region and the C2 domain. (**B**) Crystal structure of *Drosophila* Lgd (PDB ID: 6EI6) [17], encompassing its coiled-coil and C2 domains. (**C**) Superimposition of CC2D1A (green) and Lgd (blue) structures aligned by their coiled-coil domains.

In addition to the DM14 domains, the coiled-coil and C2 domains have also been structurally characterized in *Drosophila* Lgd (PDB ID: 6EI6) [17]. Structural superposition reveals a high degree of similarity between these two C2 domains, with an overall RMSD of 0.78 Å (Supplementary Figure S6A). While the core β-sandwich fold of the C2 domain is conserved between Lgd and human CC2D1A, notable differences exist in their tertiary arrangements (Figure 7). In Lgd, the coiled-coil region directly interacts with the C2 domain (Figure 7B), whereas in human CC2D1A_491–810_, these regions are separated by a long antiparallel β-sheet (Figure 7A). This β-sheet, which is absent from Lgd, functions as a structural spacer and creates distinct modular organizations. These distinct structural organizations suggest potential differences in domain-domain communication and regulatory mechanisms between the two proteins. Furthermore, in CC2D1A, Gln797 from the C-terminal loop of the C2 domain forms hydrogen bonds with Leu600 and Asn602 of the coiled-coil region, a feature not observed in Lgd. Together, these differences highlight a more intricate and tightly packed tertiary structure in human CC2D1A compared with its *Drosophila* ortholog, which might potentially reflect functional adaptations associated with nuclear repression and disease relevance.

Furthermore, the CC2D1A C2 domain harbors a conserved cluster of basic residues, Arg683, Lys696, and Lys699 (Supplementary Figure S6B). This cluster corresponds to the lipid-binding interface found in *Drosophila* Lgd (Arg689, Lys701, Lys703 in Lgd) and human CC2D1B (Arg721, Lys734, Lys736 in CC2D1B) [17]. This cluster of basic residues mediates binding to phosphatidylinositol 4,5-bisphosphate [PIns(4,5)P_2_], facilitating membrane association independent of calcium [17]. The analogous positioning of basic residues in CC2D1A suggests a similar membrane-targeting function for its C2 domain, distinct from the DNA-binding role mediated by its DM14 repeats.

## Discussion

CC2D1A is a multifunctional scaffold protein implicated in diverse physiological pathways, including transcriptional repression, cAMP-PKA signaling, and NF-κB activation, all of which are essential for neural development and homeostasis [1–7]. Loss-of-function mutations in CC2D1A have been identified as causative for autosomal recessive NSID [1,2], yet the structural basis underlying this pathogenicity has remained elusive. We determined the crystal structure of the CC2D1A_491–810_ fragment, which encompasses DM14-4, coiled-coil, and C2 domains. This region corresponds precisely to deletions found in recurrent NSID-associated alleles [1,2]. The structure reveals a conformationally integrated module that supports both nucleic acid engagement and protein complex formation.

The DM14-4-CC-C2 unit forms a structurally cohesive assembly in which each domain contributes a distinct biochemical feature: the DM14-4 domain exhibits a conserved basic patch, the coiled-coil acts as a rigid linker, and the C2 domain mediates conditional dimerization through a conserved β3–β3 interface, suggesting that C2-mediated oligomerization is conditional and potentially regulated by local cellular environment. This mechanism is reminiscent of other signaling proteins with Ca^2+^-independent C2-driven dimerization, such as PI3K-C2α and synaptotagmin [9,29].

These structural insights align with prior functional studies showing that the N-terminal DM14 domains mediate DNA binding and repress target gene expression such as 5-HT1A and DRD2 by interacting with DRE-like elements [25–27]. Our data reveal that DM14-4 shares similar electrostatic characteristics with DM14-1 to -3, supporting a broader model in which DM14 repeats serve as redundant, sequence-independent nucleic acid-binding motifs. Meanwhile, the C2 domain displays a positively charged surface resembling phospholipid-binding C2 domains and has been shown to be essential for CC2D1A-mediated NF-κB activation via interactions with TRAF2 and Ubc13 [4].

A recent comprehensive interactome study demonstrated that CC2D1A and its closely related paralog, CC2D1B, endogenously interact in the mouse brain to form a heterodimeric complex [37]. Notably, this interaction exhibits a stronger signal in the cortex than in the hippocampus, suggesting region-specific complex formation. In parallel, our crystal structure reveals that CC2D1A forms a symmetric homodimer specifically mediated by its C2 domain. Building on these two observations, we hypothesized that the C2 domain might also serve as the structural basis for CC2D1A/CC2D1B heterodimerization. To explore the structural basis of this potential interaction between CC2D1A and CC2D1B through C2 domain, we first constructed a C2 domain model of CC2D1B using AlphaFold 3. As shown in Supplementary Figure S7A, the predicted CC2D1B C2 domain exhibits extensive structural homology with the corresponding region of CC2D1A. To further provide the potential CC2D1A/CC2D1B heterodimer, we superimposed the C2 domain of CC2D1B onto one of the C2 monomer structures from our dimer structure (Supplementary Figure S7B). Our CC2D1A homodimer crystal structure reveals that the C2 domain mediates symmetric homodimerization through two critical salt-bridge pairs involving Asp697 and Lys698 (Figure 4C).

However, sequence alignment reveals that the paralog CC2D1B shares approximately 45%–50% sequence identity within this region (Figure 1B). As shown in Supplementary Figure S7C, the modeling of CC2D1A/CC2D1B heterodimer demonstrates that while the Ser735 substitution in CC2D1B (corresponding to Asp697 in CC2D1A) disrupts one of the reciprocal salt bridges, the heterodimeric interface remains energetically favorable. This stability is achieved through a single persistent salt bridge (CC2D1A-Asp697/CC2D1B-Lys736) supplemented by a compensatory hydrogen-bonding network, including CC2D1A-Lys698/CC2D1B-Gln750, Ser700/Val739, and Lys741/Lys751.

The distinct interfacial composition of the heterodimer compared with the homodimer may carry significant physiological implications. Heller et al. demonstrated that CC2D1A is uniquely enriched at the postsynaptic density (PSD), whereas CC2D1B is relatively enriched at the presynapse [37]. The loss of one reciprocal salt bridge and the emergence of a compensatory hydrogen-bonding network at the heterodimeric interface could modulate their relative binding affinities with shared partners such as CHMP4B and other ESCRT-III components, which regulate membrane trafficking in both synaptic compartments [31,38]. One possibility is that a transient CC2D1A/CC2D1B heterodimer facilitates coordinated neuronal transport, with subsequent dissociation enabling their segregation into distinct synaptic compartments; however, future biochemical studies will be needed to test this model. The structural asymmetry of the heterodimeric interface may thus contribute to the functional divergence between the two paralogs, providing a rationale for why CC2D1B cannot fully compensate for CC2D1A loss at the PSD [39,40]. Beyond synaptic localization, an equally important question is how disease-associated missense mutations [41,42] specifically disrupt CC2D1A’s local functional surfaces, such as the DNA- and lipid-binding interfaces [1,43].

Another major issue concerning CC2D1A is how disease-associated missense mutations specifically disrupt its local functional surfaces, such as the DNA- and lipid-binding interfaces [1,43]. Recently, a computational study utilized an AlphaFold-predicted model of full-length CC2D1A combined with molecular dynamics simulations to assess the impact of several NSID-associated missense mutations [41]. Notably, the key mutation sites analyzed in their study, such as Gln506Arg, Glu518Lys, Pro532Leu, and Gly781Val/Glu, all map directly into the DM14-4 and C2 domains of our CC2D1A_491–810_ structure. Our crystal structure, which demonstrates that this module forms an exceptionally stable compact unit reinforced by the antiparallel β1–β10 sheet and extensive hydrophobic core packing, is consistent with their finding that these mutations do not cause global structural collapse but instead trigger local interaction network reorganizations. This raises the possibility that NSID mutations primarily impair specific local functionalities, such as the basic patch involved in DNA binding rather than global unfolding. A critical distinction, however, is that their analyses were performed on a monomeric model, whereas our crystal structure identifies a C2-mediated homodimerization mechanism stabilized by a reciprocal β3–β3 interface, a higher-order assembly that predictive modeling alone cannot fully capture. Together, our experimental structure and their dynamic predictions are complementary, collectively providing a foundation for understanding how these mutations disrupt CC2D1A’s scaffolding and dimerization capabilities in neurodevelopmental disorders.

Finally, the architectural rigidity of the DM14-4-C2 module suggests it may act as a signal integration hub, enabling CC2D1A to coordinate transcriptional and cytoplasmic signaling axes. This is further supported by studies identifying the N-terminal region as a regulator of PDE4D in the cAMP-PKA-CREB pathway [26]. Inherited deletions that disrupt this multi-domain module would impair both DNA-dependent repression and second messenger signaling, explaining the cognitive deficits observed in NSID. Together, our findings establish the DM14-4-CC-C2 region as a functionally versatile scaffold that integrates molecular recognition, signaling specificity, and disease relevance [44].

## Supplementary Material

Supplementary Figures S1-S7 and Tables S1-S2

## Data Availability

All the data supporting the findings of the present study are available within the paper and its supplementary files. The atomic coordinates and structure factors of CC2D1A491-810 have been deposited in the Protein Data Bank (PDB) under ID code 9VHM (DOI: https://doi.org/10.2210/pdb9vhm/pdb).

## Abbreviations

CREB: cAMP response element-binding protein
CC2D1A: Coiled-coil and C2 domain containing protein 1A
CC: Coiled-coil
DM14: Drosophila melanogaster 14 domain
Lgd: Lethal (2) giant discs (*Drosophila* homolog of CC2D1A)
NSID: non-syndromic intellectual disability
PDE4D: Phosphodiesterase 4D
PKA: Protein kinase A

## References

[1] Ou X.M., Lemonde S., Jafar-Nejad H., Bown C.D., Goto A., Rogaeva A. et al. (2003) Freud-1: a neuronal calcium-regulated repressor of the 5-HT1A receptor gene. J. Neurosci. 23, 7415–7425 10.1523/JNEUROSCI.23-19-07415.2003 12917378 PMC6740452

[2] Rogaeva A., Ou X.M., Jafar-Nejad H., Lemonde S. and Albert P.R. (2007) Differential repression by freud-1/CC2D1A at a polymorphic site in the dopamine-D2 receptor gene. J. Biol. Chem. 282, 20897–20905 10.1074/jbc.M610038200 17535813

[3] Nakamura A., Naito M., Tsuruo T. and Fujita N. (2008) Freud-1/Aki1, a novel PDK1-interacting protein, functions as a scaffold to activate the PDK1/Akt pathway in epidermal growth factor signaling. Mol. Cell. Biol. 28, 5996–6009 10.1128/MCB.00114-08 18662999 PMC2546995

[4] Zhao M., Li X.D. and Chen Z. (2010) CC2D1A, a DM14 and C2 domain protein, activates NF-kappaB through the canonical pathway. J. Biol. Chem. 285, 24372–24380 10.1074/jbc.M109.100057 20529849 PMC2915672

[5] Millar A.M., Souslova T. and Albert P.R. (2012) The Freud-1/CC2D1A family: multifunctional regulators implicated in mental retardation. Latest Findings in Intellectual and Developmental Disabilities Research

[6] McMillan B.J., Tibbe C., Drabek A.A., Seegar T.C.M., Blacklow S.C. and Klein T. (2017) Structural basis for regulation of ESCRT-III complexes by Lgd. Cell Rep. 19, 1750–1757 10.1016/j.celrep.2017.05.026 28564595 PMC5528166

[7] Nakamura A., Arai H. and Fujita N. (2009) Centrosomal Aki1 and cohesin function in separase-regulated centriole disengagement. J. Cell Biol. 187, 607–614 10.1083/jcb.200906019 19948489 PMC2806580

[8] Basel-Vanagaite L., Attia R., Yahav M., Ferland R.J., Anteki L., Walsh C.A. et al. (2006) The CC2D1A, a member of a new gene family with C2 domains, is involved in autosomal recessive non-syndromic mental retardation. J. Med. Genet. 43, 203–210 10.1136/jmg.2005.035709 16033914 PMC2563235

[9] Zhao M., Raingo J., Chen Z.J. and Kavalali E.T. (2011) Cc2d1a, a C2 domain containing protein linked to nonsyndromic mental retardation, controls functional maturation of central synapses. J. Neurophysiol. 105, 1506–1515 10.1152/jn.00950.2010 21273312 PMC3075281

[10] Cheng K.H., Hung Y.C., Ling P. and Hsu K.S. (2024) Oxytocin treatment rescues irritability-like behavior in Cc2d1a conditional knockout mice. Neuropsychopharmacol. 49, 1792–1802 10.1038/s41386-024-01920-4 PMC11399130 39014123

[11] Wang Y.C., Chen C.H., Yang C.Y., Ling P. and Hsu K.S. (2023) High-fat diet exacerbates autistic-like restricted repetitive behaviors and social abnormalities in CC2D1A conditional knockout mice. Mol. Neurobiol. 60, 1331–1352 10.1007/s12035-022-03146-1 36445635

[12] Yang C.Y., Hung Y.C., Cheng K.H., Ling P. and Hsu K.S. (2021) Loss of CC2D1A in glutamatergic neurons results in autistic-like features in mice. Neurotherapeutics 18, 2021–2039 10.1007/s13311-021-01072-z 34132974 PMC8608959

[13] Satterstrom F.K., Kosmicki J.A., Wang J., Breen M.S., De Rubeis S., An J.Y. et al. (2020) Large-scale exome sequencing study implicates both developmental and functional changes in the neurobiology of autism. Cell 180, 568–584.e23 10.1016/j.cell.2019.12.036 31981491 PMC7250485

[14] Chen K.R., Chang C.H., Huang C.Y., Lin C.Y., Lin W.Y., Lo Y.C. et al. (2012) TBK1-associated protein in endolysosomes (TAPE)/CC2D1A is a key regulator linking RIG-I-like receptors to antiviral immunity. J. Biol. Chem. 287, 32216–32221 10.1074/jbc.C112.394346 22833682 PMC3442552

[15] Chen K.R., Yang C.Y., Shu S.G., Lo Y.C., Lee K.W., Wang L.C. et al. (2024) Endosomes serve as signaling platforms for RIG-I ubiquitination and activation. Sci. Adv. 10, eadq0660 10.1126/sciadv.adq0660 39504361 PMC11540011

[16] Yang D., Rismanchi N., Renvoise B., Lippincott-Schwartz J., Blackstone C. and Hurley J.H. (2008) Structural basis for midbody targeting of spastin by the ESCRT-III protein CHMP1B. Nat. Struct. Mol. Biol. 15, 1278–1286 10.1038/nsmb.1512 18997780 PMC2593743

[17] Ventimiglia L.N., Cuesta-Geijo M.A., Martinelli N., Caballe A., Macheboeuf P., Miguet N. et al. (2018) CC2D1B coordinates ESCRT-III activity during the mitotic reformation of the nuclear envelope. Dev. Cell 47, 547–563.e6 10.1016/j.devcel.2018.11.012 30513301 PMC6286407

[18] Philo J.S. (2023) SEDNTERP: a calculation and database utility to aid interpretation of analytical ultracentrifugation and light scattering data. Eur. Biophys. J. 52, 233–266 10.1007/s00249-023-01629-0 36792822

[19] Otwinowski Z. and Minor W. (1997) Processing of X-ray diffraction data collected in oscillation mode. Methods Enzymol. 276, 307–326 10.1016/S0076-6879(97)76066-X 27754618

[20] Adams P.D., Afonine P.V., Bunkoczi G., Chen V.B., Echols N., Headd J.J. et al. (2011) The Phenix software for automated determination of macromolecular structures. Methods 55, 94–106 10.1016/j.ymeth.2011.07.005 21821126 PMC3193589

[21] Powell H.R., Islam S.A., David A. and Sternberg M.J.E. (2025) Phyre2.2: a community resource for template-based protein structure prediction. J. Mol. Biol. 437, 168960 10.1016/j.jmb.2025.168960 40133783 PMC7617537

[22] Emsley P. and Cowtan K. (2004) Coot: model-building tools for molecular graphics. Acta Crystallogr. D. Biol. Crystallogr. 60, 2126–2132 10.1107/S0907444904019158 15572765

[23] Potterton L., Agirre J., Ballard C., Cowtan K., Dodson E., Evans P.R. et al. (2018) CCP4i2: the new graphical user interface to the CCP4 program suite. Acta Crystallogr. D. Struct. Biol. 74, 68–84 10.1107/S2059798317016035 29533233 PMC5947771

[24] Schrodinger, L.L.C.. (2015) The PyMOL molecular graphics system, version 1.8

[25] Lemonde S., Rogaeva A. and Albert P.R. (2004) Cell type-dependent recruitment of trichostatin A-sensitive repression of the human 5-HT1A receptor gene. J. Neurochem. 88, 857–868 10.1046/j.1471-4159.2003.02223.x 14756806

[26] Ou X.M., Jafar-Nejad H., Storring J.M., Meng J.H., Lemonde S. and Albert P.R. (2000) Novel dual repressor elements for neuronal cell-specific transcription of the rat 5-HT1A receptor gene. J. Biol. Chem. 275, 8161–8168 10.1074/jbc.275.11.8161 10713139

[27] Rogaeva A. and Albert P.R. (2007) The mental retardation gene CC2D1A/Freud-1 encodes a long isoform that binds conserved DNA elements to repress gene transcription. Eur. J. Neurosci. 26, 965–974 10.1111/j.1460-9568.2007.05727.x 17714190

[28] Krissinel E. and Henrick K. (2007) Inference of macromolecular assemblies from crystalline state. J. Mol. Biol. 372, 774–797 10.1016/j.jmb.2007.05.022 17681537

[29] Liu L., Song X., He D., Komma C., Kita A., Virbasius J.V. et al. (2006) Crystal structure of the C2 domain of class II phosphatidylinositide 3-kinase C2alpha. J. Biol. Chem. 281, 4254–4260 10.1074/jbc.M510791200 16338929

[30] Al-Tawashi A. and Gehring C. (2013) Phosphodiesterase activity is regulated by CC2D1A that is implicated in non-syndromic intellectual disability. Cell Commun. Signal. 11, 47 10.1186/1478-811X-11-47 23826796 PMC3704924

[31] Martinelli N., Hartlieb B., Usami Y., Sabin C., Dordor A., Miguet N. et al. (2012) CC2D1A is a regulator of ESCRT-III CHMP4B. J. Mol. Biol. 419, 75–88 10.1016/j.jmb.2012.02.044 22406677 PMC3433253

[32] Jumper J., Evans R., Pritzel A., Green T., Figurnov M., Ronneberger O. et al. (2021) Highly accurate protein structure prediction with AlphaFold. Nature 596, 583–589 10.1038/s41586-021-03819-2 34265844 PMC8371605

[33] Nalefski E.A. and Falke J.J. (1996) The C2 domain calcium-binding motif: structural and functional diversity. Protein Sci. 5, 2375–2390 10.1002/pro.5560051201 8976547 PMC2143302

[34] Corbalan-Garcia S. and Gomez-Fernandez J.C. (2014) Signaling through C2 domains: more than one lipid target. Biochim. Biophys. Acta 1838, 1536–1547 10.1016/j.bbamem.2014.01.008 24440424

[35] Essen L.O., Perisic O., Lynch D.E., Katan M. and Williams R.L. (1997) A ternary metal binding site in the C2 domain of phosphoinositide-specific phospholipase C-delta1. Biochemistry 36, 2753–2762 10.1021/bi962466t 9062102

[36] Troost T., Jaeckel S., Ohlenhard N. and Klein T. (2012) The tumour suppressor Lethal (2) giant discs is required for the function of the ESCRT-III component Shrub/CHMP4. J. Cell Sci. 125, 763–776 10.1242/jcs.097261 22389409

[37] Heller A.T., Bhattacharya A., Li H., Turkalj L., Thiyagarajan S., Suzuki E. et al. (2025) Interactome analysis of the CC2D1A scaffold reveals novel neuronal interactions and a postsynaptic role. bioRxiv10.1016/j.mcpro.2026.101546 PMC13052110 41765286

[38] Drusenheimer N., Migdal B., Jackel S., Tveriakhina L., Scheider K., Schulz K. et al. (2015) The mammalian orthologs of *Drosophila* Lgd, CC2D1A and CC2D1B, function in the endocytic pathway, but their individual loss of function does not affect Notch signalling. PLos Genet. 11, e1005749 10.1371/journal.pgen.1005749 26720614 PMC4697852

[39] Oaks A.W., Zamarbide M., Tambunan D.E., Santini E., Di Costanzo S., Pond H.L. et al. (2017) Cc2d1a loss of function disrupts functional and morphological development in forebrain neurons leading to cognitive and social deficits. Cereb. Cortex 27, 1670–1685 10.1093/cercor/bhw009 26826102 PMC6250986

[40] Zamarbide M., Oaks A.W., Pond H.L., Adelman J.S. and Manzini M.C. (2018) Loss of the intellectual disability and autism gene Cc2d1a and its homolog Cc2d1b differentially affect spatial memory, anxiety, and hyperactivity. Front. Genet. 9, 65 10.3389/fgene.2018.00065 29552027 PMC5840150

[41] Abuelrub A., Erol I., Nalbant Bingol N., Ozemri Sag S., Temel S.G. and Durdagi S. (2025) Computational analysis of CC2D1A missense mutations: insight into protein structure and interaction dynamics. ACS Chem. Neurosci. 16, 3665–3681 10.1021/acschemneuro.4c00570 39791913 PMC12498394

[42] Ma A.C.H., Mak C.C.Y., Yeung K.S., Pei S.L.C., Ying D., Yu M.H.C. et al. (2020) Monoallelic mutations in CC2D1A suggest a novel role in human heterotaxy and ciliary dysfunction. Circ. Genom. Precis. Med. 13, e003000 10.1161/CIRCGEN.120.003000 33196317 PMC7748040

[43] Gallagher C.M. and Knoblich J.A. (2006) The conserved c2 domain protein lethal (2) giant discs regulates protein trafficking in *Drosophila*. Dev. Cell 11, 641–653 10.1016/j.devcel.2006.09.014 17084357

[44] Yeh Y.H., Lin M.G., Sun X.H. and Hsiao C.D. (2026) Structure of CC2D1A (coiled-coil and C2 domain containing - 1A). PDB 10.2210/pdb9vhm/pdb

[45] Robert X. and Gouet P. (2014) Deciphering key features in protein structures with the new ENDscript server. Nucleic. Acids. Res. 42, W320–W324 10.1093/nar/gku316 24753421 PMC4086106

