# Supplementary material for: Structural characterization of the human CC2D1A fragment associated with non-syndromic intellectual disability (NSID)
Source: Biosci Rep. 2026 May 21;46(6):BSR20253955. doi: 10.1042/BSR20253955 (PMC13199796; doi:10.1042/BSR20253955)
Supplement: Supplementary Figures S1-S7 and Tables S1-S2 [file BSR-2025-3955_T_supp.pdf]

Supplementary data

Supplementary Table S1. X-ray diffraction data and refinement statistics of human CC2D1A$_{491\text{-}810}$

Crystal	Human CC2D1A$_{491\text{-}810}$
**Data collection statistics**	
Source	NSRRC-TPS 05A
Wavelength (Å)	0.97625
Space group	$P2_12_12_1$
Resolution (Å)	2.3
Unit cell parameters	
a, *b*, *c* (Å)	55.38 / 70.26 / 201.45
Redundancy of reflection	6.0 (4.9)[a]
Completeness (%), overall	96.5 (85.6)
$I/\sigma(I)$, overall	29.16 (2.4)
R_{merge}[b] (%), overall	7.3 (55.7)
$CC_{1/2}$, overall	0.965 (0.856)
**Refinement statistics**	
Resolution (Å)	27.4- 2.3
R-factor[c]/R_{free}[d] (%)	21.5 / 27.0
Number of reflections used	33645
Number of residues	640
Number of atoms	
Protein	5102
Water	119
B-factor (Å^2)	
Protein	58.94
Water	49.10
RMSD bond lengths (Å)	0.003
RMSD bond angles (°)	0.58
PDB ID[e]	9VHM

[a] Values in parentheses are for the highest-resolution shell.
[b] $R_{merge}=\Sigma|I-\langle I\rangle|/\Sigma I$, where I is the observed intensity and $\langle I\rangle$ is the average intensity from multiple observations of symmetry-related reflections.
[c] $R=\Sigma|F_{obs}-F_{calc}|/\Sigma F_{obs}$, where F_{obs} and F_{calc} are the observed and calculated structure factor amplitudes, respectively.
[d] R_{free} was calculated with 5% of the total number of reflections randomly omitted from the refinement.
[e] Protein data bank identifiers for co-ordinates.

Supplementary Table S2: Sequence identity in human CC2D1A DM14 1-4.

	DM14-1	DM14-2	DM14-3	DM14-4
DM14-1	100	31.2	32.7	32.1
DM14-2	31.2	100	34.0	44.2
DM14-3	32.7	34.0	100	35.6
DM14-4	32.1	44.2	35.6	100

Supplementary Figures

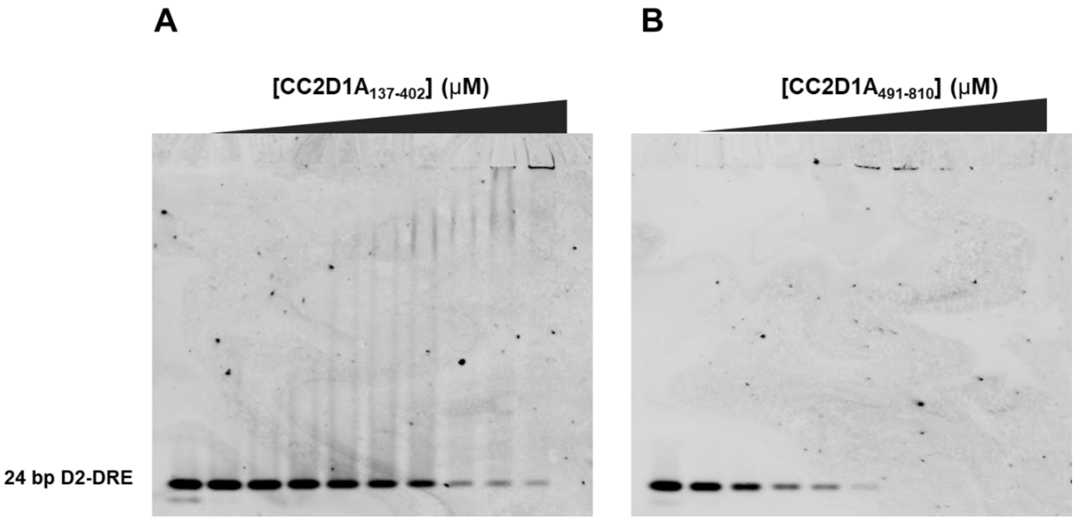

Supplementary Figure S1: DNA binding ability of (A) CC2D1A$_{137\text{-}402}$ and (B) CC2D1A$_{491\text{-}810}$.

Representative EMSA in which increasing concentrations (0.4-100 μM) of CC2D1A were incubated with 10 nM 5'end Cy3-labeled 24bp D2-DRE dsDNA.

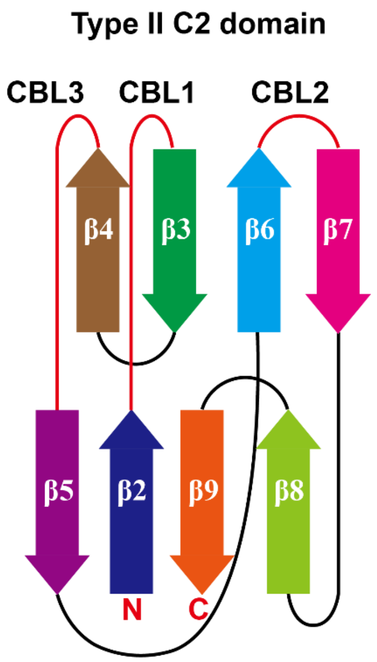

Supplementary Figure S2: Topology diagram of the CC2D1A C2 domain.

Schematic representation of the secondary structure topology of the CC2D1A C2 domain, classified as a Type II C2 domain. The canonical calcium-binding loops (CBL1, CBL2 and CBL3), typically found between β2-β3 and β6-β7 in classical C2 domains, are indicated but do not contain conserved acidic residues in CC2D1A.

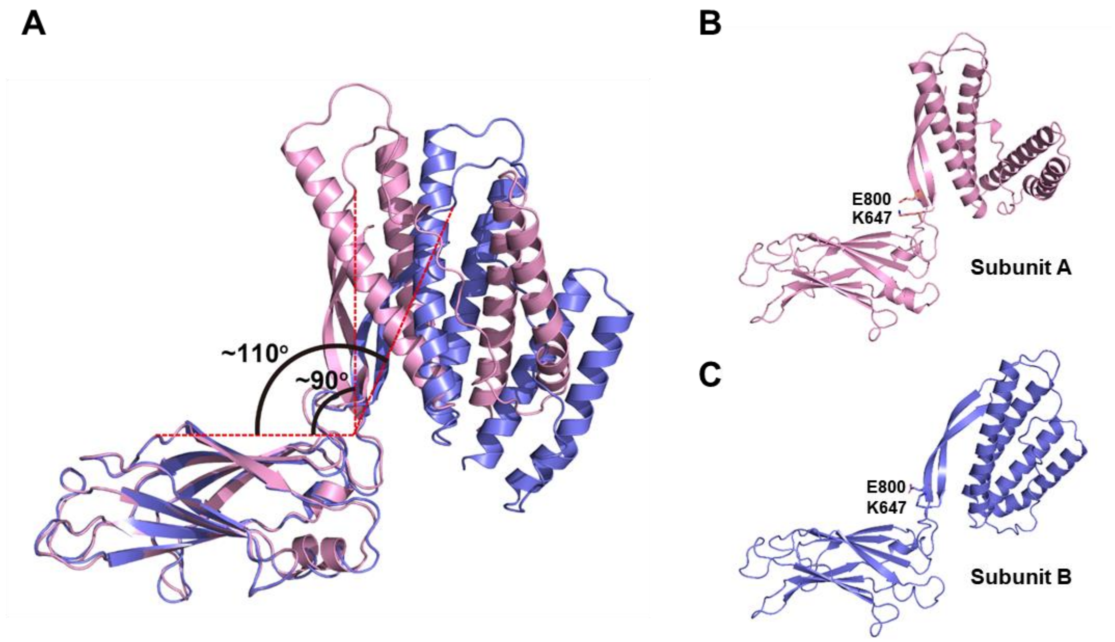

Supplementary Figure S3: Superimposition of the Cα atoms of the C2 domains between Subunit A and Subunit B from the CC2D1A$_{491-810}$ crystal structure.

(A) Superimposition of subunit A (pink) and subunit B (blue), aligned by their C2 domains, reveals a distinct difference in the orientation of the coiled-coil region. The angle between the coiled-coil and C2 domains is approximately 90° in subunit A and 110° in subunit B. **(B)** Structure of subunit A colored in pink, showing the coiled-coil and C2 domains. The inter-subunit salt bridge pair Lys647-Glu800 is labeled. **(C)** Structure of subunit B colored in blue, showing the corresponding Lys647-Glu800.

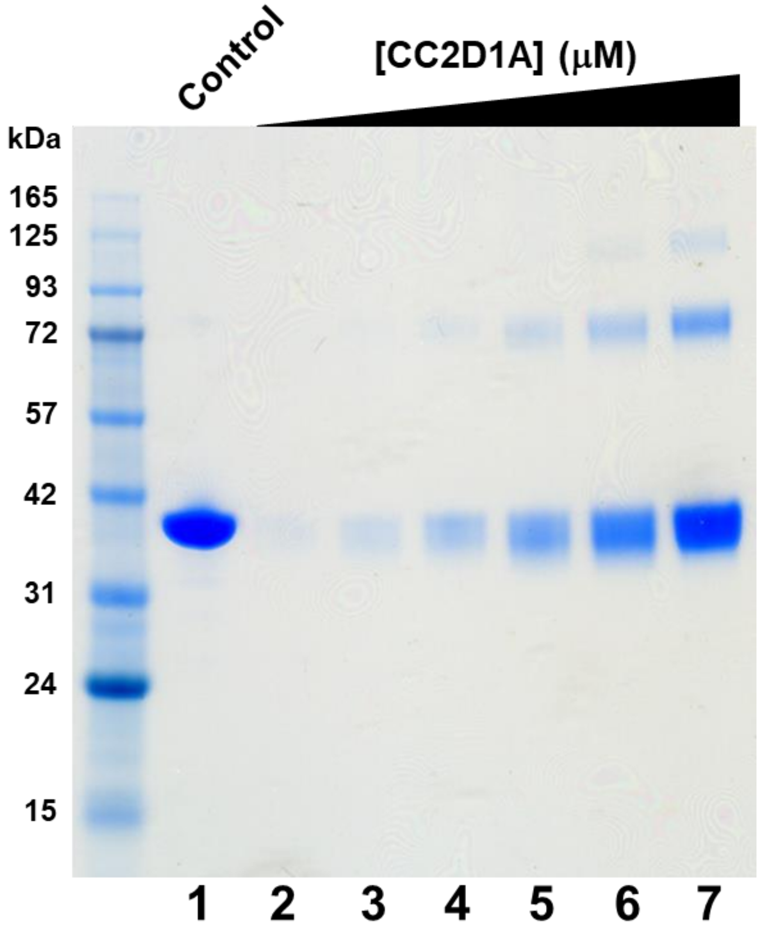

Supplementary Figure S4: Concentration-dependent DSS cross-linking of CC2D1A$_{491\text{-}810}$.

CC2D1A$_{\mathbf{491\text{-}810}}$ was incubated with 0.5 mM DSS cross-linker at increasing protein concentrations (0.25-10 μM) to assess the oligomerization state. Lane 1 shows the untreated control (10 μM CC2D1A$_{491\text{-}810}$ without DSS), which migrates as a monomer. Lanes 2 to 7 correspond to CC2D1A$_{\mathbf{491\text{-}810}}$ concentrations of 0.25, 0.5, 1, 2.5, 5 and 10 μM, respectively. Cross-linking products were analyzed in 4-12% SDS-PAGE gels.

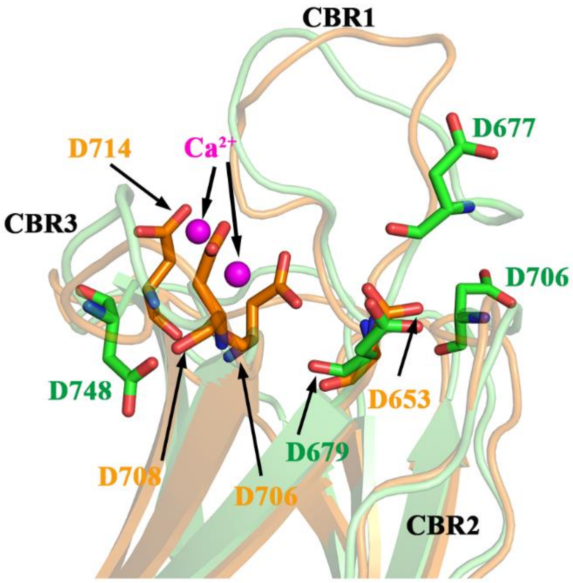

Supplementary Figure S5: Structural comparison of Ca^{2+}-binding region in CC2D1A and canonical C2 domains.

Superposition of the C2 domain of CC2D1A (green) with the type II Ca^{2+}-binding C2 domain of phospholipase Cδ (PLCδ, orange). The Ca^{2+} ions (magenta spheres) are derived from the PLCδ structure, and Ca^{2+}-binding C2 domains highlights key differences in the architecture of Ca^{2+}-binding regions CBR1-CBR3.

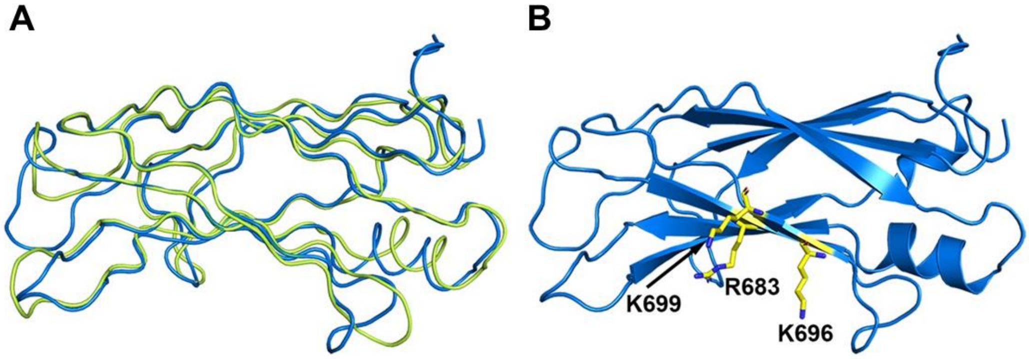

Supplementary Figure S6: Structural comparison of the C2 domains from human CC2D1A and *Drosophila* Lgd.

(**A**) Backbone superposition of the C2 domains from human CC2D1A (blue) and Drosophila Lgd (green), showing a high degree of structural conservation in the β-sandwich core. (**B**) Ribbon representation of the CC2D1A C2 domain highlighting a conserved basic patch composed of Arg683, Lys696, and Lys699 (yellow sticks).

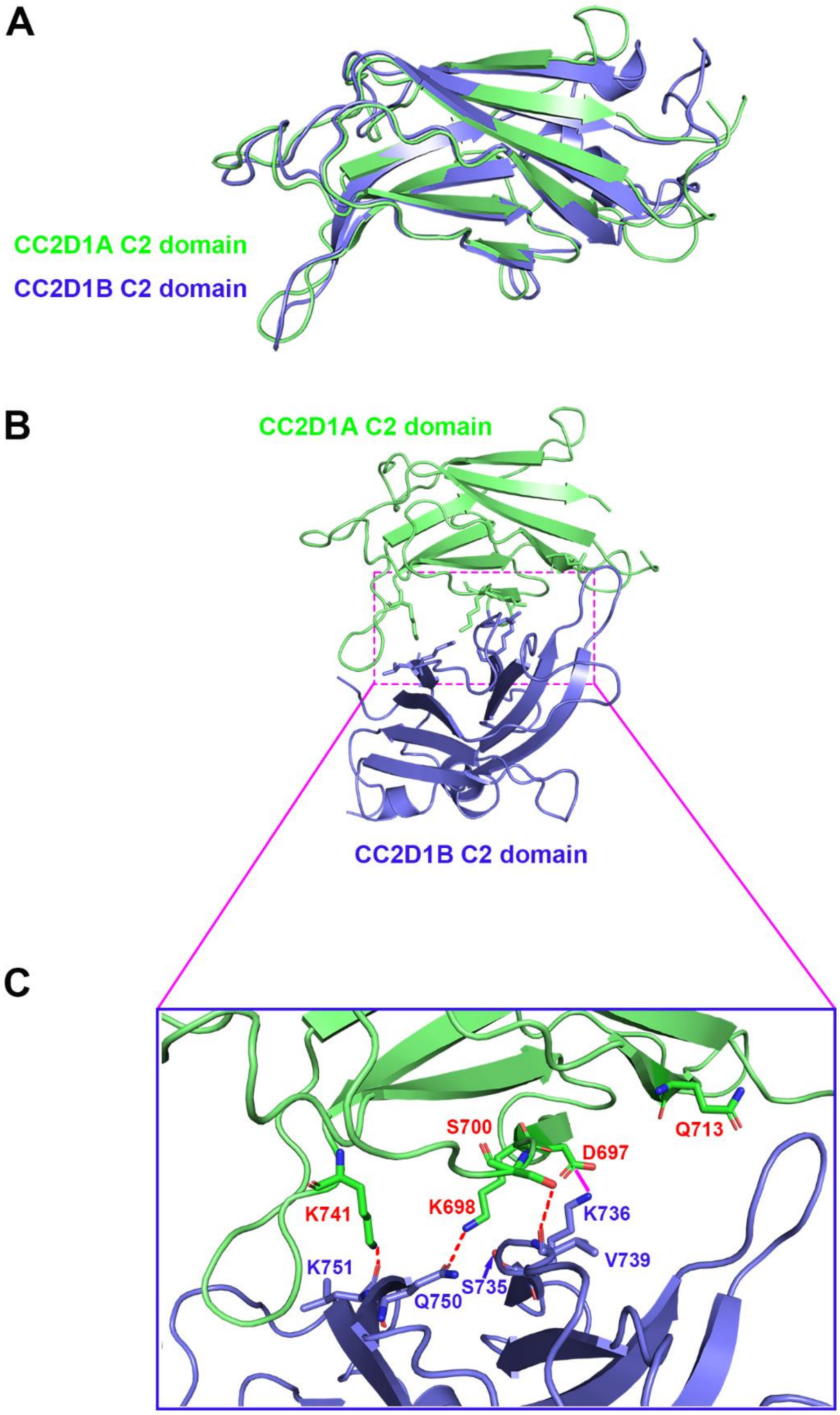

Supplementary Figure S7: Structural model of the CC2D1A and CC2D1B heterodimer.

To model the CC2D1A/CC2D1B heterodimer, the sequence of the human CC2D1B C2 domain (residues 528-850) corresponding to the CC2D1A fragment (residues

491-810) was used to generate a structural prediction via AlphaFold 3. The resulting CC2D1B model was then superimposed onto one monomer within the existing experimental CC2D1A homodimer. To resolve potential steric clashes and optimize the heterodimeric interface, the complex underwent structural refinement using the geometry minimization module within the PHENIX package. **(A)** Structural superimposition of the AlphaFold-predicted CC2D1B C2 domain (colored in blue) and the C2 domain of the CC2D1A crystal structure (colored in green), demonstrating their high structural homology. **(B)** Overall structural model of the CC2D1A/CC2D1B heterodimeric assembly, which is primarily mediated by the reciprocal interaction between the C2 domains. **(C)** Close-up view of the specific amino acid interactions at the C2-C2 heterodimeric interface. The key residues contributing to the intermolecular salt bridge and the compensatory hydrogen-bonding network are labeled and shown as sticks.
